# Thyroid function in the subacute phase of traumatic brain injury: a potential predictor of post-traumatic neurological and functional outcomes

**DOI:** 10.1007/s40618-021-01656-8

**Published:** 2021-08-05

**Authors:** C. Mele, L. Pagano, D. Franciotta, M. Caputo, A. Nardone, G. Aimaretti, P. Marzullo, V. Pingue

**Affiliations:** 1 Department of Clinical-Surgical, Diagnostic and Pediatric Sciences, University of Pavia, Pavia, Italy; 2 Division of Endocrinology, Diabetology and Metabolism, Department of Medical Sciences, University of Turin, Turin, Italy; 3 IRCCS Ospedale Policlinico San Martino, Genoa, Italy; 4 Department of Health Sciences, University of Piemonte Orientale, Novara, Italy; 5 Neurorehabilitation and Spinal Unit, Istituti Clinici Scientifici Maugeri SPA SB, Institute of Pavia, IRCCS, Pavia, Italy; 6 Department of Translational Medicine, University of Piemonte Orientale, Novara, Italy; 7 Division of General Medicine, IRCCS Istituto Auxologico Italiano, Ospedale San Giuseppe, Verbania, Italy

**Keywords:** Thyroid, Epilepsy, Traumatic brain injury, Outcome

## Abstract

**Purpose:**

That thyroid hormones exert pleiotropic effects and have a contributory role in triggering seizures in patients with traumatic brain injury (TBI) can be hypothesized. We aimed at investigating thyroid function tests as prognostic factors of the development of seizures and of functional outcome in TBI.

**Methods:**

This retrospective study enrolled 243 adult patients with a diagnosis of mild-to-severe TBI, consecutively admitted to our rehabilitation unit for a 6-month neurorehabilitation program. Data on occurrence of seizures, brain imaging, injury characteristics, associated neurosurgical procedures, neurologic and functional assessments, and death during hospitalization were collected at baseline, during the workup and on discharge. Thyroid function tests (serum TSH, fT4, and fT3 levels) were performed upon admission to neurorehabilitation.

**Results:**

Serum fT3 levels were positively associated with an increased risk of late post-traumatic seizures (LPTS) in post-TBI patients independent of age, sex and TBI severity (OR = 1.85, CI 95% 1.22–2.61, *p* < 0.01). Measured at admission, fT3 values higher than 2.76 pg/mL discriminated patients with late post-traumatic seizures from those without, with a sensitivity of 74.2% and a specificity of 60.9%. Independently from the presence of post-traumatic epilepsy and TBI severity, increasing TSH levels and decreasing fT3 levels were associated with worse neurological and functional outcome, as well as with higher risk of mortality within 6 months from the TBI event.

**Conclusions:**

Serum fT3 levels assessed in the subacute phase post-TBI are associated with neurological and functional outcome as well as with the risk of seizure occurrence. Further studies are needed to investigate the mechanisms underlying these associations.

## Introduction

Traumatic brain injury (TBI) is a recognized public health problem and constitutes a frequent cause of disability and even death in adults. The incidence of TBI has been increasing worldwide during the last decades, reaching a rate between 134 and 618 persons per 100.000 per year and a hospitalization rate of about 12% [1, 2]. TBI is associated with several pathophysiological mechanisms, which underlie heterogeneous clinical manifestations. The primary damage caused by the mechanical injury can instigate a cascade of inflammatory, metabolic and biochemical alterations leading to secondary injury. These processes have been associated with the onset of chronic neurological and endocrine complications [3], which can significantly impact overall morbidity and mortality [4, 5].

Long-term consequence of TBI potentially includes post-traumatic seizures (PTS). Based on the time of onset, PTS have been classified as early post-traumatic seizures (EPTS), which occur within 7 days from injury and are characterized by a temporarily decreased seizure threshold following the primary injury [6, 7], and late post-traumatic seizures (LPTS), which occur within weeks or months following the traumatic event and are associated with persistent neurobiological changes triggered by the secondary injury cascade [7]. The high risk of recurrent seizures following a single LPTS leads to consider LPTS as an epileptic condition. Therefore, the term LPTS is often used interchangeably with post-traumatic epilepsy (PTE) [7, 8].

Despite the growing evidence about the potential role of neuroinflammation and of metabolic alterations in influencing both early and late post-traumatic epileptogenesis, the pathogenetic mechanisms underlying these conditions have not been fully elucidated yet.

A potential involvement of thyroid hormones (THs) in the pathogenesis of epilepsy has been proposed [9]. THs play an essential role in the development and maintenance of brain functions [10]. Such role in the brain homeostasis emerges from the neurological complications of hypothyroidism in both infants and adults [11]. THs exert genomic and non-genomic effects on mitochondrial function [12] and neurotransmission [13], as well as modulate the development and function of GABAergic interneurons [14], which partake in different microcircuits recruited in epilepsy [15]. Moreover, both excess and deficiency of THs affect the oxidative brain status through modulation of antioxidant enzymes [16]. Clinically, reduced serum levels of THs with thyrotropin (TSH) in the normal reference range have been observed in most patients with severe injuries and are referred to as nonthyroidal illness (NTI) [17]. Although the molecular mechanisms underlying these neurofunctional effects remain still largely unknown, a role for THs on inhibitory and excitatory neuronal circuits affecting brain electrical activity can be hypothesized, thus contributing to trigger seizures and, more in general, functional outcomes in TBI patients [18].

To date, there are no clinical studies exploring the potential association between thyroid function, seizures and post-TBI outcome. Therefore, this study was designed to investigate the role of THs as predictive biomarkers of the occurrence of seizures and relating functional outcomes in a large cohort of patients with TBI.

## Methods

### Patients

**Fig. 1:**
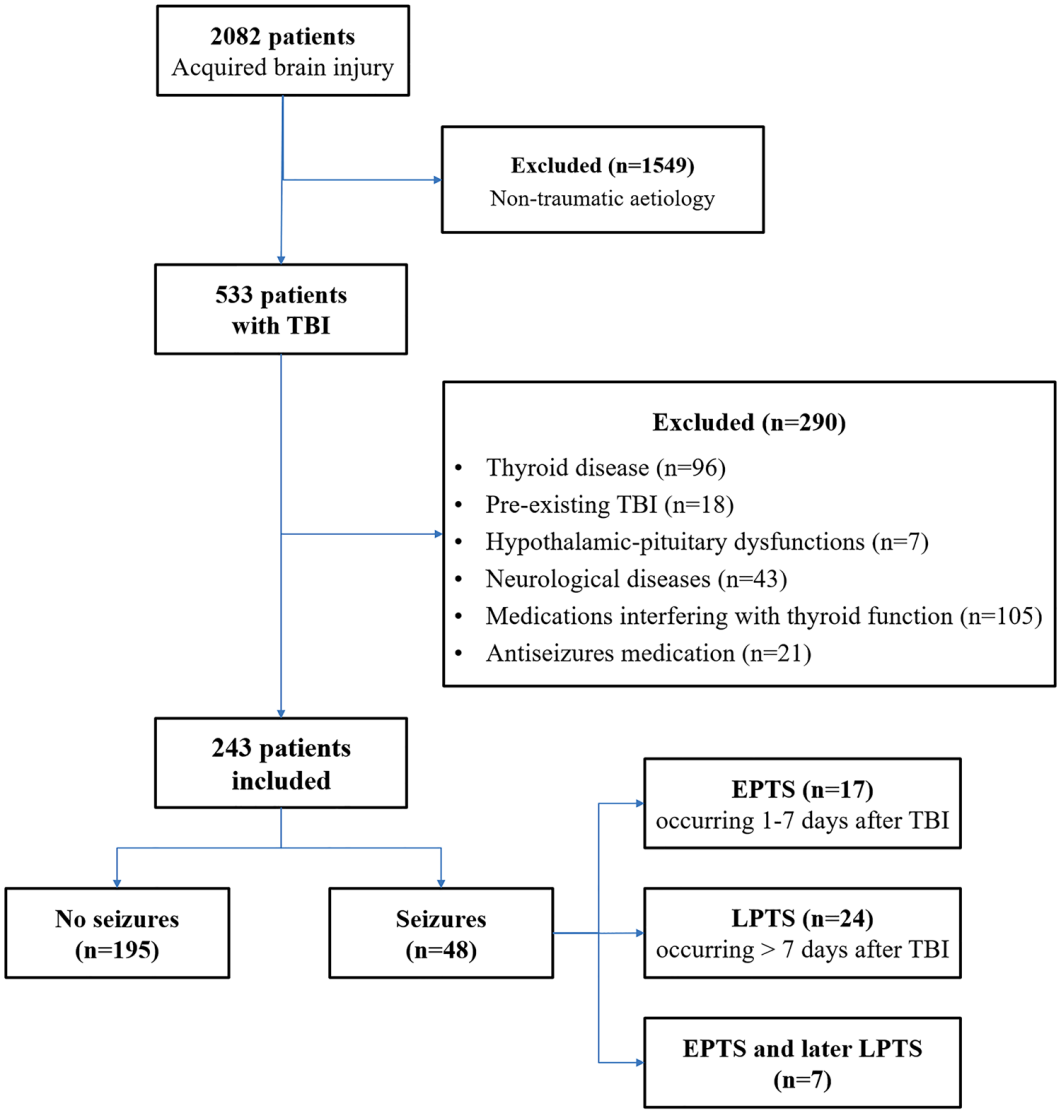
Flowchart of the study’s participants. *EPTS* early post-traumatic seizures, *LPTS* late post-traumatic seizures, *TBI* traumatic brain injury

A total of 2082 adult patients with the diagnosis of acquired brain injury were admitted to the Neurorehabilitation Unit of the Istituti Clinici Scientifici (ICS) Maugeri of Pavia between January 1, 2009 and December 31, 2018; 1549 were excluded because of a non-traumatic aetiology, and 290 did not meet the inclusion criteria (Fig. 1).

Eligibility criteria included age ≥ 18 years, diagnosis of mild-to-severe TBI based on the Glasgow Coma Scale (GCS) as followingly detailed, admission to a hospital emergency unit within 24 h after the traumatic event and admission to our rehabilitation unit for a 6-month neurorehabilitation programme within 14 days following the event. Exclusion criteria were the presence of known thyroid disease, pre-existing TBI, known hypothalamic–pituitary dysfunctions or neurological diseases including epilepsy, use of levothyroxine (LT4) or triiodothyronine (T3) and/or use of medications potentially interfering with thyroid function and use of antiepileptic drugs (AED) before the injury. Therefore, 243 patients with mild-to-severe TBI were included.

The study design was conformed to the ethical guidelines of the Declaration of Helsinki and was approved by the local Ethical Committee ICS Maugeri (#2214 CE). The participants or authorized representatives signed a written informed consent.

### Methods

The data were retrieved in the electronic hospital records at baseline, during the workup and on discharge, and included the following variables: sex, medical history, age, occurrence of seizures, brain imaging, injury characteristics, fracture site, presence of subarachnoid haemorrhage, associated neurosurgical procedures (craniotomy, cranioplasty), neurologic and functional assessments, use and type of AED and death during hospitalization. Thyroid function tests (TSH, fT4, fT3) were performed upon admission to neurorehabilitation (on average, 14 ± 3 days after TBI).

All participants underwent a 6-month inpatient neurorehabilitation program consisting of individual 3-h daily treatment cycles, 6 days per week inclusive of physiotherapy, occupational therapy, speech therapy, cognitive training, nutrition assistance, as well as psychological and social support.

Seizures occurring during acute and rehabilitation period were classified into two categories defined taking into account the time elapsed from injury: early, if occurring 1–7 days after TBI (EPTS); late, if occurring > 7 days after TBI (LPTS) [19].

The Marshall computed tomography (CT) classification was used to categorize TBI into six classes according to the degree of swelling as determined by basal cistern compression and midline shift, as well as the presence and size of focal lesions (Table 1) [20].

**Table 1:** Marshall’s computed tomography (CT) classification

Marshall class	Definition
Diffuse injury I	No intracranial pathology seen with CT
Diffuse injury II	Cisterns are present with midline shift of 0–5 mm and/or lesions/densities present; no high-or mixed density lesions > 25 cm^3^; may include bone fragments and foreign bodies
Diffuse injury III (swelling)	Cisterns compressed or absent with midline shift of 0–5 mm; no high or mixed density lesions > 25 cm^3^
Diffuse injury IV (shift)	Midline shift > 5 mm; no high or mixed density lesions > 25 cm^3^
Evacuated mass lesion (V)	Any lesion surgically evacuated
Non-evacuated mass lesion (VI)	High or mixed density lesion > 25 cm^3^, not surgically evacuated

The severity of TBI on admission was assessed according to the GCS. It is a standardized system for assessing the degree of neurological impairment and to identify the seriousness of injury in relation to outcome, which involves three determinants: eye opening, verbal responses and motor response or movement. These determinants are evaluated separately according to a numerical value that indicates the level of consciousness and the degree of dysfunction. Total scores range from 15 to 3. Patients are considered to have experienced a “mild” brain injury when their score is from 13 to 15. A score from 9 to 12 indicates a “moderate” brain injury, and a score equal to 8 or less reflects a “severe” brain injury [21]. Rehabilitation outcomes were evaluated through the Functional Independence Measure (FIM) scale, an 18-item measurement tool that explores individual's physical, psychological and social function [22, 23]. The tool is used to assess the patient's level of disability as well as change in patient status in response to rehabilitation or medical intervention [24].

Finally, GCS and FIM scale were administered on admission and at discharge to evaluate neurological and rehabilitation outcomes, respectively.

### Thyroid function tests

Serum samples were assayed for fT4, fT3 and TSH using an automated chemiluminescence assay system (Immulite 2000; DPC, Los Angeles, CA). The principle of the method is a two-site, solid-phase chemiluminescent immunometric assay (TSH) or competitive immunoassay (fT4 and fT3). The reference ranges are 0.4–4.0 µIU/mL for TSH, 0.8–1.8 ng/dL for fT4, and 1.8–4.2 pg/mL for fT3.

### Statistical analysis

Values are expressed as means ± standard deviation (SD), or absolute number and percentage. Data were tested for normality of distribution by the Shapiro–Wilk test and log-transformed when needed, to correct for skewness. For comparative analysis, ANOVA between groups was used. Pearson’s correlation analysis and *χ*^2^ were used to identify significant associations between variables of interest. Univariate and multinomial logistic regression analyses were performed to evaluate the association between thyroid function and the presence of epilepsy, clinical and radiological characteristics of TBI as well as mortality. An analysis of the receiver operating characteristic (ROC) curve and the area under the curve (AUC) was undertaken to assess the best cutoff for fT3 to discriminate between patients at higher risk and patients at lower risk of developing seizures. Statistical significance was set at 5%. Statistical analyses were performed using SPSS version 21 (Somers, NY, USA).

## Results

### Clinical and radiological characteristics of TBI

A summary of clinical and radiological characteristics of population with TBI is reported in Table 2. The male-to-female ratio was 3.5:1. Patients aged ≤ 65 years were 57.6% of cases. Approximately, half of the patients presented a skull fracture, most of which (50.0%) affected the neurocranium compound. With regard to brain injury, 69.9% of patients suffered from multiple site lesions mostly involving the frontal lobe. Subarachnoid haemorrhage was detected in 40.3% of cases. Neurosurgical procedures had been performed in 53.1% of patients and included craniotomy and cranioplasty in 39.5% and 13.6% of cases, respectively.

**Table 2:** Clinical and radiological characteristics of patients with traumatic brain injury (TBI) as a whole and subgrouped according to the Glasgow Coma Scale (GCS)

Variables	Whole population (*n* = 243)	TBI severity (GCS) (data available for 211 patients)			
	*N* (%)	Mild 18 (8.5)	Moderate 51 (24.2)	Severe 142 (67.3)	*p* value
		*N* (%)	*N* (%)	*N* (%)	
Sex					
Males	189 (77.8)	9 (50.0)	43 (84.3)	111 (78.2)	**0.01**
Females	54 (22.2)	9 (50.0)	8 (15.7)	31 (21.8)	
Age (years)					
≤ 65	140 (57.6)	3 (16.7)	28 (54.9)	93 (65.5)	**0.0003**
> 65	103 (42.4)	15 (83.3)	23 (45.1)	49 (34.5)	
Adapted Marshall classification*					
Diffuse injury I	11 (4.6)	1 (5.5)	3 (5.9)	2 (1.4)	0.20
Diffuse injury II	60 (24.9)	1 (5.5)	13 (25.5)	36 (25.4)	0.17
Diffuse injury III (swelling)	44 (18.2)	6 (33.4)	8 (15.7)	24 (16.9)	0.21
Diffuse injury IV (shift)	52 (21.6)	3 (16.7)	16 (31.4)	28 (19.7)	0.19
Evacuated lesion	74 (30.7)	7 (38.9)	11 (21.6)	52 (36.6)	0.13
Non evacuated lesion	0 (0.0)	0 (0.0)	0 (0.0)	0 (0.0)	–
Subarachnoid haemorrhage					
Yes	98 (40.3)	5 (27.8)	24 (47.1)	59 (41.5)	0.36
No	145 (59.7)	13 (72.2)	27 (52.9)	83 (58.5)	
Lobar localization^§^					
Frontal	26 (15.0)	2 (13.3)	6 (15.4)	15 (14.8)	0.97
Parietal	4 (2.3)	0 (0.0)	0 (0.0)	2 (2.0)	0.61
Temporal	20 (11.6)	2 (13.3)	2 (5.1)	14 (13.9)	0.39
Occipital	2 (1.2)	0 (0.0)	1 (2.6)	1 (1.0)	0.66
Multiple	121 (69.9)	11 (73.4)	30 (76.9)	69 (68.3)	0.33
Cranial fractures					
Yes	118 (48.6)	7 (38.9)	25 (49.0)	75 (52.8)	0.52
No	125 (51.4)	11 (61.1)	26 (51.0)	67 (47.2)	
Fracture site					
Splanchnocranium	27 (22.9)	2 (28.6)	4 (16.0)	20 (26.7)	0.48
Skull base	10 (8.5)	2 (28.6)	3 (12.0)	5 (6.7)	0.15
Compound skull fracture	59 (50.0)	3 (42.8)	15 (60.0)	35 (46.7)	0.92
Depressed skull fracture	20 (16.9)	0 (0.0)	3 (12.0)	14 (18.7)	0.27
From blunt body	2 (1.7)	0 (0.0)	0 (0.0)	1 (1.3)	0.80
Craniotomy					
Yes	96 (39.5)	6 (33.3)	18 (35.3)	67 (47.2)	0.23
No	147 (60.5)	12 (66.7)	33 (64.7)	75 (52.8)	
Cranioplasty					
Yes	33 (13.6)	4 (22.2)	6 (11.8)	21 (14.8)	0.56
No	210 (86.4)	14 (77.8)	45 (88.2)	121 (85.2)	
PTS					
Yes	48 (19.8)	3 (16.7)	9 (17.6)	30 (21.1)	0.81
No	195 (80.2)	15 (83.3)	42 (82.4)	112 (78.9)	
EPTS					
Yes	17 (7.0)	1 (5.5)	4 (7.8)	9 (6.3)	0.92
No	226 (93.0)	17 (94.5)	47 (92.2)	133 (93.7)	
LPTS					
Yes	24 (9.9)	1 (5.5)	5 (9.8)	16 (11.3)	0.70
No	219 (90.1)	17 (94.5)	46 (90.2)	126 (88.7)	
EPTS + LPTS					
Yes	7 (2.9)	1 (5.5)	0 (0.0)	5 (3.5)	0.33
No	236 (97.1)	17 (94.5)	51 (100)	137 (96.5)	
AED					
No therapy	140 (57.6)	12 (66.6)	28 (54.9)	79 (55.6)	0.65
Prophylactic therapy	64 (26.3)	3 (16.7)	15 (29.4)	41 (28.9)	0.53
Therapy for crisis	39 (16.1)	3 (16.7)	8 (15.7)	22 (15.5)	0.99
Mortality within 6 months					
Yes	35 (14.4)	4 (22.2)	7 (13.7)	22 (15.5)	0.69
No	208 (85.6)	14 (77.8)	44 (86.3)	120 (84.5)	

Data available for the *whole population*: *241 patients, ^§^173 patients; *Mild TBI*: ^§^15 patients; *Moderate TBI*: ^§^39 patients; *Severe TBI*: ^§^101 patients. Comparison between group was performed by Chi square analysis. Significant differences are shown in bold characters

*ASM* antiseizure medication, *GCS* Glasgow Coma Scale, *EPTS* early post-traumatic seizures, *LPTS* late post-traumatic seizures, *PTS* post-traumatic seizure

According to GCS assessed in the acute phase, TBI was classified as severe in 67.3%, moderate in 24.2% and mild in 8.5% of patients. Clinical and radiological characteristics of TBI were comparable between the three classes of TBI severity, whereas patients with moderate and severe TBI were significantly younger (*p* = 0.0003) and with a higher prevalence of male when compared to patients with mild TBI (*p* = 0.01). No differences in the prevalence of seizures were found between the classes of TBI severity.

After TBI, prophylactic AED therapy was started in 64 patients (26.3%), of whom 6 (9.4%) subsequently developed seizures. Such therapy was prescribed to 39 patients (16.1%) and after that they had developed seizures. Most patients (75.7%) were treated with levetiracetam (II generation AED).

During the observation period from acute care hospitalization to inpatient rehabilitation, seizures occurred in 48 patients (19.8%). Overall, EPTSs were documented in 17 cases (7.0%), LPTS in 24 cases (9.9%), whereas 7 patients (2.9%) first presented EPTS and then LPTS.

### Post-TBI thyroid function

The results of thyroid function testing in the population as a whole and across the three classes of TBI severity are summarized in Table 3. Serum TSH and fT4 levels were comparable between classes of TBI severity, whereas fT3 levels were significantly lower in moderate and severe TBI in comparison with mild TBI (*p* < 0.05).

**Table 3:** Thyroid function variables in the population as a whole and subgrouped according to TBI severity classification (mild, moderate and severe) on the basis of GCS

Variables	Whole population (*n* = 243)	TBI classification^&^ (GCS)			
	Mean ± SD	Mild (*n* = 18)	Moderate (*n* = 51)	Severe (*n* = 142)	*p* value
		Mean ± SD	Mean ± SD	Mean ± SD	
TSH (µUI/mL)	2.0 ± 1.8	1.8 ± 1.3	2.1 ± 2.1	2.1 ± 1.4	0.404
fT4 (ng/dL)	1.3 ± 0.4	1.2 ± 0.2	1.3 ± 0.6	1.3 ± 0.4	0.781
fT3 (pg/mL)	2.7 ± 0.7	**2.9 ± 0.5**	**2.6 ± 0.6**	**2.7 ± 0.7**	**0.048**

Data available from the whole population: ^&^211 patients. Comparison between groups was performed by ANOVA. Significant differences are shown in bold characters

When TSH levels were divided according to the quartile ranges (I quartile: TSH < 1.05 µUI/mL; II quartile: 1.05 ≤ TSH < 1.74 µUI/mL; III quartile: 1.74 ≤ TSH < 2.40 µUI/mL; IV quartile: TSH > 2.40 µUI/mL), patients were evenly distributed within TSH quartiles, with fT4 and fT3 levels at the lower limit of the reference range or reduced in 19.0% and 44.0%, respectively. There were no differences in fT4 and fT3 levels within TSH ranges.

Thyroid function variables were not associated with age, sex, characteristics and type of TBI. Moreover, TSH, fT4 and fT3 were not correlated to each other (data not shown).

### Thyroid function and seizures

Analysis of thyroid function variables according to the presence or absence of PTS in the population as a whole showed overall significantly higher levels of fT3 in patients who developed LPTS than those without PTS (Table 4). No significant differences were found for TSH and fT4 levels between subgroups of patients.

**Table 4:** Post-TBI thyroid function in patients without and with PTS and, among these, with EPTS or LPTS

Variables	No PTS (195 cases)	PTS (48 cases)	EPTS (17 cases)	LPTS (24 cases)
	Mean ± SD	Mean ± SD	Mean ± SD	Mean ± SD
TSH (µUI/mL)	2.0 ± 1.6	2.0 ± 1.3	1.7 ± 1.2	1.9 ± 0.9
fT4 (ng/dL)	1.3 ± 0.4	1.3 ± 0.2	1.3 ± 0.2	1.3 ± 0.3
fT3 (pg/mL)	**2.6 ± 0.7**	2.7 ± 0.8	2.4 ± 0.7	**3.0 ± 0.7**^a^

Comparison between groups was performed by ANOVA. Significant differences are shown in bold characters. Significant differences between patients without and with epilepsy or EPTS or LPTS are expressed as ^a^*p* < 0.01

**Fig. 2:**
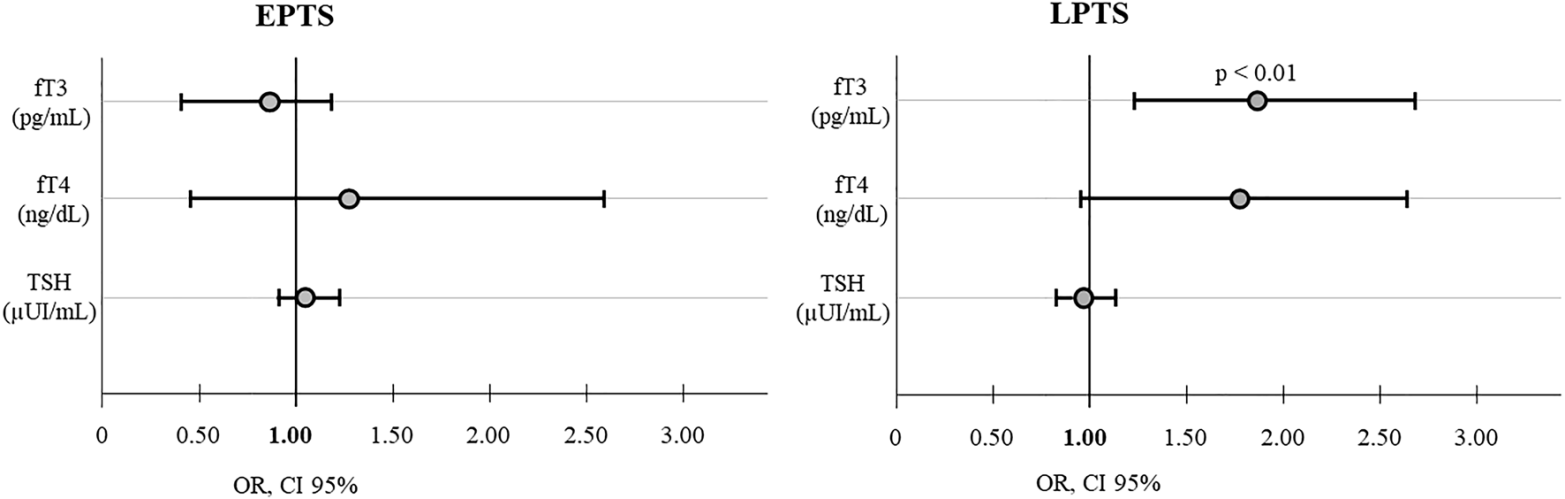
Odds ratios (ORs) for the association between thyroid function and EPTS or LPTS

A multinomial logistic regression analysis was conducted to evaluate the association between thyroid function variables and the risk of seizures. Independently from age, sex and TBI severity, fT3 levels were directly associated with an increased risk of LPTS onset (OR = 1.85, CI 95% 1.22–2.61, *p* < 0.01) (Fig. 2). TSH and fT4 levels were not associated with EPTS or LPTS (not shown).

**Fig. 3:**
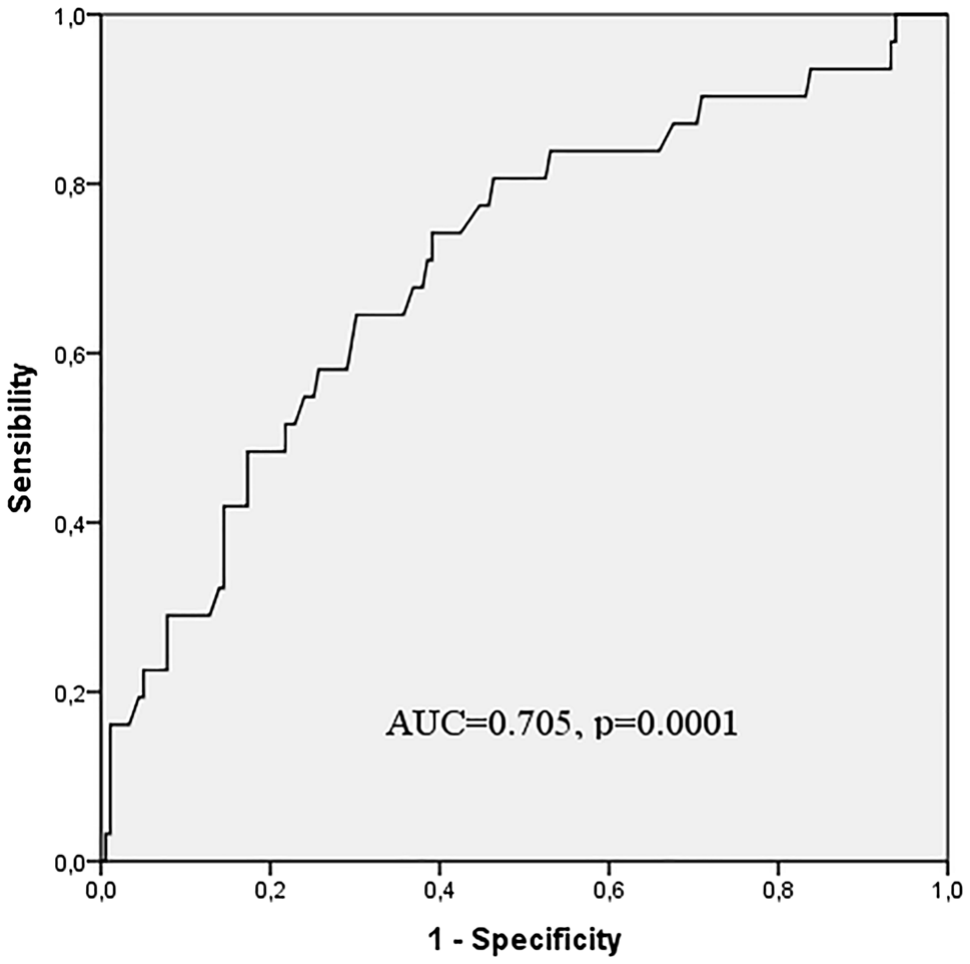
ROC curve for fT3 levels in the process of discriminating patients with a higher risk of LPTS onset

Analysis of the ROC curve (Fig. 3) showed that, in our study population, fT3 represented a potentially predictive parameter for identifying patients at higher risk of developing LPTS (AUC = 0.705, CI 95% 0.602–0.808, *p* = 0.0001). Taking fT3 values higher than 2.76 pg/mL as cutoff, fT3 levels revealed the ability to discriminate patients presenting with LPTS from those without, with a sensitivity of 74.2% and a specificity of 60.9%.

With regard to the use of AED, there were no differences in thyroid function variables between users and nonusers (Table 5), also when the three TBI groups were analyzed separately.

**Table 5:** Post-TBI thyroid function in users and nonusers of antiepileptic drugs

Variables	Users of prophylactic AED (64 patients)	Nonusers of prophylactic AED (179 patients)	*p* value
	Mean ± SD	Mean ± SD	
TSH (µUI/mL)	2.2 ± 2.0	1.9 ± 1.7	0.31
fT4 (ng/dL)	1.2 ± 0.4	1.3 ± 0.3	0.70
fT3 (pg/mL)	2.7 ± 0.8	2.6 ± 0.7	0.16

Comparison between groups was performed by ANOVA

*AED* antiepileptic drug

### Thyroid function and neurological/functional outcome

Correlation analysis between thyroid function parameters, neurological and functional outcomes showed that increasing TSH levels and decreasing fT3 levels were associated with worst neurological and functional outcomes in terms of GCS and FIM, respectively (Table 6). No associations were found between fT4 levels and neurological or functional outcomes.

**Table 6:** Pearson’s correlation analysis between thyroid function variables and neurological as well as functional outcome in the study populations as a whole

Variables	TSH (µUI/mL)		fT4 (ng/dL)		fT3 (pg/mL)	
	*r*	*p*	*r*	*p*	*r*	*p*
GCS T0	0.08	0.19	**− **0.12	0.08	0.11	0.14
**GCS T1**	**− 0.16**	**0.02**	**− **0.08	0.29	**0.32**	** < 0.0001**
**ΔGCS**	**− 0.20**	**0.001**	0.03	0.65	**0.18**	**0.009**
FIM T0	**− **0.03	0.66	0.13	0.09	**− **0.21	0.78
**FIM T1**	**− 0.14**	**0.04**	0.05	0.56	**0.15**	**0.04**
**ΔFIM**	**− 0.17**	**0.02**	**− **0.03	0.72	**0.20**	**0.009**

Significant differences are shown in bold characters

*GCS* Glasgow Coma Scale, *FIM* Functional Independence Measure, *T0* on admission in neurorehabilitation, *T1* on discharge

Significant correlations obtained on the entire dataset remained after controlling for the presence or the absence of seizures and for TBI severity.

### Thyroid function and mortality post-TBI

Mortality within 6 months of TBI was documented in 35 patients (14.4%), without a significant difference in prevalence between the three classes of TBI severity. Independent of the presence of seizures, clinical or radiological characteristics of TBI, and TBI severity, the multinomial logistic regression analysis showed that increasing TSH levels and decreasing fT3 levels were associated with higher risk of mortality within 6 months from TBI event (TSH: OR = 1.3, CI 95% 1.09–1.45, *p* < 0.01; fT3: OR = 0.21, CI 95% 0.11–0.41, *p* < 0.0001).

## Discussion

The present study evaluated the association of thyroid function with the development of seizures and the neurological and functional outcomes in patients with TBI. Our results showed that fT3 levels were directly associated with an increased risk of LPTS onset independent of age, sex and TBI severity. On the other hand, increasing TSH levels and decreasing fT3 levels were associated with worst neurological and functional outcome, as well as with higher risk of mortality within 6 months from TBI event independent of PTS and TBI severity.

A transient reduction of serum fT4 and fT3 levels, with TSH in the normal range, has been observed in TBI and referred to as NTI [17]. NTI following brain injury was initially described by Woolf et al. [25] in a cohort of 66 patients with severe TBI, and was subsequently investigated by Malekpour et al. [26] in a prospective study on 72 patients. Our results confirm these findings in a cohort of patients with mild-to-severe TBI. In fact, we found that approximately 90% harbored normal TSH levels and approximately half of them had reduced fT3 levels. It is known that acute illnesses, including TBI, are able to induce multiple alterations in thyroid function parameters in patients without previously ascertained thyroid diseases [17]. Despite these changes in THs, there is scarce evidence of a direct thyroid dysfunction in this setting. This hormone pattern could be hypothetically viewed as the result of a physiological adaptive mechanism aiming to downregulate the metabolic activity and energy balance in condition of acute injury as a result of the following potential mechanisms: (1) deregulation of deiodinase activity resulting in an impairment of T4 to T3 conversion [27], leading to increased levels of reverse-T3 [28]; (2) alterations in pituitary TSH secretion originating from TRH inhibition from cortical centers and/or abnormalities in TSH secretory rhythms [29]; and (3) alterations in the affinity of binding proteins for thyroid hormone due to critical illness, which can significantly contribute to determine a reduction of T4 and T3 levels [17]. Although serum TSH levels are usually normal during the early post-acute phase of TBI, we were unable to find a correlation between TSH, fT4 and fT3. We hypothesize that this circumstance possibly reflects either a central derangement of pituitary TSH secretion, or an alteration of systemic metabolic homeostasis in the immediate post-acute phase of TBI. In fact, post-TBI pathophysiological mechanisms lead to loss of body mass, negative nitrogen balance, dysglycaemia and cerebral metabolic dysfunction. In this setting, the changes in serum THs levels could be mainly the result of alterations in the peripheral metabolism of the THs, in the binding of TH to transport protein, in receptor binding, and in intracellular uptake, rather than a response to an alteration of TSH levels [30].

Our data analysis suggests a potential involvement of patients’ post-TBI thyroid status on their subsequent risk of manifesting epileptic disorders. TBI represents an important risk factor for adult epilepsy [31]. Epilepsy is a neurological disorder characterized by a continuous rise in neuronal excitability, which leads to recurrent and spontaneous seizures resulting in an altered function and morphology of neuronal cells [32]. Although the exact pathogenesis of PTE remains still unknown, a relationship between dysfunction of blood–brain barrier (BBB) following TBI and epilepsy has also been documented, both in animal and human studies [33]. Our results, in agreement with other clinical studies, show that there is no association between the occurrence of seizures and the severity of TBI within 6 months [34–36]. The main reason for this lack of association lies in the etiopathogenesis of post-traumatic seizures. Post-TBI neuroinflammation, which occurs regardless of the severity of the trauma, exerts a pivotal role in influencing epileptogenesis and long-term neurological complications in mild-to-severe TBI [37–40]. Some authors hypothesized that THs could contribute to the pathogenesis of epilepsy [9]. Unlike TSH and fT4, our results showed that fT3 levels were directly associated with an increased risk of LPTS onset in patients with TBI independent of age, sex and TBI severity. Despite being within the normal range, a fT3 cutoff value of 2.76 pg/mL was apparently able to discriminate patients at higher risk of developing seizures from those at lower risk. It is known that the increased transcellular permeability of the BBB allows the extravasation of immune cells, proteins and solutes from the cerebral vasculature into the interstitial space, promoting abnormal neuronal excitability which could contribute to epileptogenesis [41–43]. In this context, the altered permeability of BBB could also allow an abnormal entry of THs from the bloodstream to the brain tissue. Molecular studies showed that THs, particularly T3, exert a pivotal role in normal mitochondrial biogenesis and their alterations cause mitochondrial dysfunction and oxidative stress [44, 45], which are in turn related to experimental epileptogenesis and human epilepsy [46]. From a clinical viewpoint, some studies demonstrated that THs dysregulation, either hyperthyroidism or hypothyroidism, affects antioxidant/oxidant balance, thus promoting ROS generation and oxidative stress, which could play a potential role in the pathogenesis of epilepsy [9]. Some authors hypothesized that hyperthyroidism could more frequently induce epileptogenesis than hypothyroidism and case reports have suggested an association between hyperthyroidism and seizures, with correction of thyrotoxicosis leading to resolution of seizures [47]. To determine the prevalence of seizure caused by the thyrotoxic state, Song et al. retrospectively evaluated the prevalence and clinical features of seizures in 3382 patients with hyperthyroidism. However, after exclusion of patients with seizures and/or with a history of epilepsy or other CNS impairments, only the 0.2% of patients were found to have acute seizures probably related to thyrotoxicosis [48].

In addition to the increased permeability of BBB, another mechanism that could potentially explain the seizures-inducing effect of circulating T3 regards deiodinase 3 (D3) local activity. A regional expression of D3 activity has been demonstrated in specific areas of the human CNS, and a critical role for D3 has been suggested in the regulation of local T3 content in concert with other enzymes [49]. In physiological conditions, D3 protects the brain from high doses of T3 by reducing its effects [50, 51]. However, in response to injury, a deregulation of D3 has been observed in CNS [52], thus altering local fT3 levels and promoting epileptogenesis. Overall, whether this THs-mediated mechanism could contribute to post-TBI epileptogenesis remains to be investigated.

For what concern EPTS, we did not find any association between EPTS occurrence and fT3 levels. Although our study is not able to determine the mechanisms underlying the association between serum fT3 levels and post-TBI seizures, it is well known that EPTS and LPTS have different aetiologies. In fact, EPTS occurrence is caused by mechanisms related to primary injury that temporarily lower the seizure threshold, whereas LPTS onset is associated with persistent neurobiological changes attributed to secondary injury and in particular neuroinflammatory alterations [6, 7, 19]. As mentioned above, neuroinflammation is able to alter the permeability of the blood brain barrier (BBB), thus likely impairing the passage of THs within the CNS and favouring epileptogenesis. Therefore, while fT3 levels in the acute phase may not influence the onset of EPTS, relatively higher fT3 in this early phase may instead represent a potential predictor of LPTS.

It is known that TBI represents an important cause of death and disability in young adults [1]. The role of THs on neurological outcomes and mortality after TBI is debated. Malekpour et al. [26] showed that reduced serum T4 and T3 levels were associated with worse GCS and increased mortality in TBI patients, whereas TSH levels were unrelated to their clinical outcomes. On the contrary, Chioléro et al. [53] observed that TSH and T3 levels were correlated with the severity of TBI and mortality. Our results seem to agree with the latter. In fact, in our cohort we did not find any association between fT4 levels and neurological outcome or mortality, whereas we observed that increasing TSH and declining fT3 levels were associated with worst neurological and functional outcomes, as well as with a higher risk of mortality within 6 months following TBI. The evidence of an association between THs and neurological or functional outcomes led some authors to investigate on the potential role of THs replacement therapy in improving these outcomes, with controversial results [17, 54–56].

Our study has some limitations. First, the study is aimed to find associations, without insights into mechanisms, which require ad hoc investigations. Second, we did not assess the evaluation of the thyroid function during and at the end of the rehabilitation process, and this hampers a full interpretation of the herein observed abnormalities. Third, being a retrospective study, we did not evaluate the presence of nodular goiter in our cohort. Nodular goiter is a condition typically characterized by autonomously functioning thyroid cell, which might partly explain the fact that thyroid function was not fully regulated by the hypothalamic–pituitary–thyroid axis.

In conclusion, serum fT3 levels assessed in the subacute phase post-TBI are associated with neurological and functional outcome as well as with the risk of seizure occurrence. Further studies are needed to investigate the mechanisms underlying these associations.

## Data Availability

The datasets generated during and/or analysed during the current study are available from the corresponding author on reasonable request.

## References

[1] Peeters W, van den Brande R, Polinder S, Brazinova A, Steyerberg EW, Lingsma HF, Maas AI (2015). Epidemiology of traumatic brain injury in Europe. Acta Neurochir (Wien).

[2] Nguyen R, Fiest KM, McChesney J, Kwon CS, Jette N, Frolkis AD, Atta C, Mah S, Dhaliwal H, Reid A, Pringsheim T, Dykeman J, Gallagher C (2016). The international incidence of traumatic brain injury: a systematic review and meta-analysis. Can J Neurol Sci.

[3] Corrigan JD, Hammond FM (2013). Traumatic brain injury as a chronic health condition. Arch Phys Med Rehabil.

[4] Harrison-Felix C, Pretz C, Hammond FM, Cuthbert JP, Bell J, Corrigan J, Miller AC, Haarbauer-Krupa J (2015). Life expectancy after inpatient rehabilitation for traumatic brain injury in the United States. J Neurotrauma.

[5] Zaloshnja E, Miller T, Langlois JA, Selassie AW (2008). Prevalence of long-term disability from traumatic brain injury in the civilian population of the United States, 2005. J Head Trauma Rehabil.

[6] Hunt RF, Boychuk JA, Smith BN (2013). Neural circuit mechanisms of post-traumatic epilepsy. Front Cell Neurosci.

[7] Fisher RS, Acevedo C, Arzimanoglou A, Bogacz A, Cross JH, Elger CE, Engel J, Forsgren L, French JA, Glynn M, Hesdorffer DC, Lee BI, Mathern GW, Moshé SL, Perucca E, Scheffer IE, Tomson T, Watanabe M, Wiebe S (2014). ILAE official report: a practical clinical definition of epilepsy. Epilepsia.

[8] Haltiner AM, Temkin NR, Dikmen SS (1997). Risk of seizure recurrence after the first late posttraumatic seizure. Arch Phys Med Rehabil.

[9] Tamijani SM, Karimi B, Amini E, Golpich M, Dargahi L, Ali RA, Ibrahim NM, Mohamed Z, Ghasemi R, Ahmadiani A (2015). Thyroid hormones: possible roles in epilepsy pathology. Seizure.

[10] de Escobar GM, Obregón MJ, del Rey FE (2004). Maternal thyroid hormones early in pregnancy and fetal brain development. Best Pract Res Clin Endocrinol Metab.

[11] Schroeder AC, Privalsky ML (2014). Thyroid hormones, t3 and t4, in the brain. Front Endocrinol (Lausanne).

[12] Yuen AW, Sander JW (2011). Impaired mitochondrial energy production: the basis of pharmacoresistance in epilepsy. Med Hypotheses.

[13] Calzà L, Fernandez M, Giardino L (2010). Cellular approaches to central nervous system remyelination stimulation: thyroid hormone to promote myelin repair via endogenous stem and precursor cells. J Mol Endocrinol.

[14] Westerholz S, de Lima AD, Voigt T (2010). Regulation of early spontaneous network activity and GABAergic neurons development by thyroid hormone. Neuroscience.

[15] Righes Marafiga J, Vendramin Pasquetti M, Calcagnotto ME (2020). GABAergic interneurons in epilepsy: More than a simple change in inhibition. Epilepsy Behav.

[16] Das K, Chainy GB (2004). Thyroid hormone influences antioxidant defense system in adult rat brain. Neurochem Res.

[17] Farwell AP (2013). Nonthyroidal illness syndrome. Curr Opin Endocrinol Diabetes Obes.

[18] Hoffmann G, Dietzel ID (2004). Thyroid hormone regulates excitability in central neurons from postnatal rats. Neuroscience.

[19] Agrawal A, Timothy J, Pandit L, Manju M (2006). Post-traumatic epilepsy: an overview. Clin Neurol Neurosurg.

[20] Marshall LF, Marshall SB, Klauber MR, MvB C, Eisenberg HM, Jane JA, Luerssen TG, Marmarou A, Foulkes MA (1991). A new classification of head injury based on computerized tomography. J Neurosurg.

[21] Teasdale G, Jennett B (1974). Assessment of coma and impaired consciousness. A practical scale. Lancet.

[22] Linacre JM, Heinemann JW, Wright BD, Granger CV, Hamilton BB (1994). The structure and stability of the functional independence measure. Arch Phys Med Rehabil.

[23] Mackintosh S (2009). Functional independence measure. Aust J Physiother.

[24] Heinemann AW, Linacre JM, Wright BD, Hamilton BB, Granger C (1993). Relationships between impairment and physical disability as measured by the functional independence measure. Arch Phys Med Rehabil.

[25] Woolf PD, Lee LA, Hamill RW, McDonald JV (1988). Thyroid test abnormalities in traumatic brain injury: correlation with neurologic impairment and sympathetic nervous system activation. Am J Med.

[26] Malekpour B, Mehrafshan A, Saki F, Malekmohammadi Z, Saki N (2012). Effect of posttraumatic serum thyroid hormone levels on severity and mortality of patients with severe traumatic brain injury. Acta Med Iran.

[27] Bianco AC, Kim BW, Braverman LE, Cooper DS (2013). Intracellular pathways of iodothyronine metabolism/implications of deiodinations for thyroid hormone action. Werner and Ingbar’s The Thyroid.

[28] Huang SA, Bianco AC (2008). Reawakened interest in type III iodothyronine deiodinase in critical illness and injury. Nat Clin Pract Endocrinol Metab.

[29] Haddady S, Farwell AP, Irwin RS, Rippe JM (2011). Non-thyroidal illness and the management of thyroid disorders in the Intensive Care Unit. Intensive care medicine.

[30] Economidou F, Douka E, Tzanela M, Orfanos S, Kotanidou A, Rajendram R, Preedy VR, Patel VB (2015). Thyroid function in critical illness. Diet and nutrition in critical care.

[31] Lowenstein DH (2009). Epilepsy after head injury: an overview. Epilepsia.

[32] Engelborghs S, D'Hooge R, De Deyn PP (2000). Pathophysiology of epilepsy. Acta Neurol Belg.

[33] Oby E, Janigro D (2006). The blood-brain barrier and epilepsy. Epilepsia.

[34] Ritter AC, Wagner AK, Fabio A, Pugh MJ, Walker WC, Szaflarski JP, Zafonte RD, Brown AW, Hammond FM, Bushnik T, Johnson-Greene D, Shea T, Krellman JW, Rosenthal JA, Dreer LE (2016). Incidence and risk factors of posttraumatic seizures following traumatic brain injury: a Traumatic Brain Injury Model Systems Study. Epilepsia.

[35] Strazzer S, Pozzi M, Avantaggiato P, Zanotta N, Epifanio R, Beretta E, Formica F, Locatelli F, Galbiati S, Clementi E, Zucca C (2016). Late post-traumatic epilepsy in children and young adults: impropriety of long-term antiepileptic prophylaxis and risks in tapering. Paediatr Drugs.

[36] Pingue V, Mele C, Nardone A (2021). Post-traumatic seizures and antiepileptic therapy as predictors of the functional outcome in patients with traumatic brain injury. Sci Rep.

[37] Sharma R, Leung WL, Zamani A, O'Brien TJ, Casillas Espinosa PM, Semple BD (2019). Neuroinflammation in post-traumatic epilepsy: pathophysiology and tractable therapeutic targets. Brain Sci.

[38] Zimmermann LL, Martin RM, Girgis F (2017). Treatment options for posttraumatic epilepsy. Curr Opin Neurol.

[39] Lucke-Wold BP, Nguyen L, Turner RC, Logsdon AF, Chen YW, Smith KE, Huber JD, Matsumoto R, Rosen CL, Tucker ES, Richter E (2015). Traumatic brain injury and epilepsy: underlying mechanisms leading to seizure. Seizure.

[40] Webster KM, Sun M, Crack P, O'Brien TJ, Shultz SR, Semple BD (2017). Inflammation in epileptogenesis after traumatic brain injury. J Neuroinflamm.

[41] Tomkins O, Shelef I, Kaizerman I, Eliushin A, Afawi Z, Misk A, Gidon M, Cohen A, Zumsteg D, Friedman A (2008). Blood-brain barrier disruption in post-traumatic epilepsy. J Neurol Neurosurg Psychiatry.

[42] Shlosberg D, Benifla M, Kaufer D, Friedman A (2010). Blood-brain barrier breakdown as a therapeutic target in traumatic brain injury. Nat Rev Neurol.

[43] Dadas A, Janigro D (2019). Breakdown of blood brain barrier as a mechanism of post-traumatic epilepsy. Neurobiol Dis.

[44] Halliwell B, Gutteridge JMC (2001). Free radicals in biology & medicine.

[45] Martinez B, Rodrigues TB, Gine E, Kaninda JP, Perez-Castillo A, Santos A (2009). Hypothyroidism decreases the biogenesis in free mitochondria and neuronal oxygen consumption in the cerebral cortex of developing rats. Endocrinology.

[46] Pauletti A, Terrone G, Shekh-Ahmad T, Salamone A, Ravizza T, Rizzi M, Pastore A, Pascente R, Liang LP, Villa BR, Balosso S, Abramov AY, van Vliet EA, Del Giudice E, Aronica E, Patel M, Walker MC, Vezzani A (2019). Targeting oxidative stress improves disease outcomes in a rat model of acquired epilepsy. Brain.

[47] Koch L (2010). Prevalence of thyrotoxicosis-related seizures. Nat Rev Endocrinol.

[48] Song TJ, Kim SJ, Kim GS, Choi YC, Kim WJ (2010). The prevalence of thyrotoxicosis-related seizures. Thyroid.

[49] Santini F, Pinchera A, Ceccarini G, Castagna M, Rosellini V, Mammoli C, Montanelli L, Zucchi V, Chopra IJ, Chiovato L (2001). Evidence for a role of the type III-iodothyronine deiodinase in the regulation of 3,5,3'-triiodothyronine content in the human central nervous system. Eur J Endocrinol.

[50] Freitas BC, Gereben B, Castillo M, Kalló I, Zeöld A, Egri P, Liposits Z, Zavacki AM, Maciel RM, Jo S, Singru P, Sanchez E, Lechan RM, Bianco AC (2010). Paracrine signaling by glial cell-derived triiodothyronine activates neuronal gene expression in the rodent brain and human cells. J Clin Invest.

[51] Galton VA, Schneider MJ, Clark AS, St Germain DL (2009). Life without thyroxine to 3,5,3'-triiodothyronine conversion: studies in mice devoid of the 5'-deiodinases. Endocrinology.

[52] Simonides WS, Mulcahey MA, Redout EM, Muller A, Zuidwijk MJ, Visser TJ, Wassen FW, Crescenzi A, da-Silva WS, Harney J, Engel FB, Obregon MJ, Larsen PR, Bianco AC, Huang SA (2008). Hypoxia-inducible factor induces local thyroid hormone inactivation during hypoxic-ischemic disease in rats. J Clin Invest.

[53] Chioléro RL, Lemarchand-Béraud T, Schutz Y, de Tribolet N, Bayer-Berger M, Freeman J (1988). Thyroid function in severely traumatized patients with or without head injury. Acta Endocrinol (Copenh).

[54] Shulga A, Blaesse A, Kysenius K, Huttunen HJ, Tanhuanpää K, Saarma M, Rivera C (2009). Thyroxin regulates BDNF expression to promote survival of injured neurons. Mol Cell Neurosci.

[55] Crupi R, Paterniti I, Campolo M, Di Paola R, Cuzzocrea S, Esposito E (2013). Exogenous T3 administration provides neuroprotection in a murine model of traumatic brain injury. Pharmacol Res.

[56] Kaptein EM, Beale E, Chan LS (2009). Thyroid hormone therapy for obesity and nonthyroidal illnesses: a systematic review. J Clin Endocrinol Metab.

